# The Impact of Obesity and Lifestyle on the Immune System and Susceptibility to Infections Such as COVID-19

**DOI:** 10.3389/fnut.2020.597600

**Published:** 2020-11-19

**Authors:** Daan L. de Frel, Douwe E. Atsma, Hanno Pijl, Jacob C. Seidell, Pieter J. M. Leenen, Willem A. Dik, Elisabeth F. C. van Rossum

**Affiliations:** ^1^Department of Cardiology, Leiden University Medical Center, Leiden, Netherlands; ^2^Department of Endocrinology, Leiden University Medical Center, Leiden, Netherlands; ^3^Department of Health Sciences, VU Medical Center, Amsterdam, Netherlands; ^4^Department of Immunology, Erasmus MC, University Medical Center Rotterdam, Rotterdam, Netherlands; ^5^Laboratory Medical Immunology, Department of Immunology, Erasmus MC, University Medical Center Rotterdam, Rotterdam, Netherlands; ^6^Division of Clinical Immunology, Department of Internal Medicine, Erasmus MC, University Medical Center Rotterdam, Rotterdam, Netherlands; ^7^Division of Endocrinology, Department of Internal Medicine, Erasmus MC, University Medical Center Rotterdam, Rotterdam, Netherlands; ^8^Obesity Center CGG, Erasmus MC, University Medical Center Rotterdam, Rotterdam, Netherlands

**Keywords:** obesity, diet, COVID-19, lifestyle, immune system

## Abstract

**Background:** COVID-19 is a global challenge to healthcare. Obesity is common in patients with COVID-19 and seems to aggravate disease prognosis. In this review we explore the link between obesity, chronic disease, lifestyle factors and the immune system, and propose societal interventions to enhance global immunity.

**Search Strategy and Selection Criteria:** We performed three literature searches using the keywords (1) coronavirus AND comorbidities, (2) comorbidities AND immune system, and (3) lifestyle factors AND immune system. Results were screened for relevance by the main author and a total of 215 articles were thoroughly analyzed.

**Results:** The relationship between obesity and unfavorable COVID-19 prognosis is discussed in light of the impact of chronic disease and lifestyle on the immune system. Several modifiable lifestyle factors render us susceptible to viral infections. In this context, we make a case for fostering a healthy lifestyle on a global scale.

**Conclusions:** Obesity, additional chronic disease and an unhealthy lifestyle interactively impair immune function and increase the risk of severe infectious disease. In adverse metabolic and endocrine conditions, the immune system is geared toward inflammation. Collective effort is needed to ameliorate modifiable risk factors for obesity and chronic disease on a global scale and increase resistance to viruses like SARS-CoV-2.

## Introduction

In recent months the entire globe has been affected by coronavirus disease 2019 (COVID-19), caused by severe acute respiratory syndrome coronavirus 2 (SARS-CoV-2) (1). Worldwide more than 11 million cases of COVID-19 have been detected until July 2020 and over 500,000 cases have proved fatal (2) Despite serious efforts, no effective vaccine has been developed yet. But, even without a vaccine, the impact of subsequent waves of SARS-CoV-2 spreading can most likely be decreased. Among patients with COVID-19, obesity, hypertension, cardiovascular disease (CVD) and type 2 diabetes (T2D) are noticeable risk factors for hospitalization, ICU admission, and death. Apparently, chronic disease is linked with unfavorable outcome in COVID-19. As appropriate lifestyle intervention effectively ameliorates cardiometabolic conditions, public health could play a pivotal role in the battle against SARS-CoV-2 (3). The aim of this review is to summarize the current understanding of the links between lifestyle, metabolic syndrome, obesity, and increased susceptibility to viral infections. In addition, we aim to investigate whether public health interventions can affect resistance to viral infections.

## Search Strategy and Selection Criteria

Three separate literature searches were performed in PubMed, EMBASE, and Google Scholar through April 2020 to May 2020. We considered English-language (systematic) review articles and meta-analyses found using the following term combinations: (1) “Coronavirus” and “diabetes/hypertension/obesity/cardiovascular disease/metabolic syndrome” yielding 1,132 articles on April 29, 2020. (2) “Immune system” and “diabetes/hypertension/obesity/cardiovascular disease/metabolic syndrome” yielding 1,364 articles on May 17, 2020. (3) “Immune system” and “lifestyle” yielding 753 results on June 4, 2020. The complete search strategies can be found in Supplementary Material. References lists of identified publications were also screened for relevant studies. If multiple articles were found per subject, preference went to clarity, relevance, and novelty. A total of 215 articles (17, 151, and 47 for search strategies 1, 2, and 3, respectively) were scrutinized for this review.

## Obesity and Cardiometabolic Diseases are Related to Worse Outcome of COVID-19

The Chinese Center for Disease Control reports higher COVID-19 fatality rates in patients with comorbidities as compared with patients without (4). The fatality rate for patients with COVID-19 without comorbidities was 0.9%, whereas fatality rates in patients with diabetes (7.3%), CVD (10.5%), and hypertension (6.0%) are clearly higher (4). American and European studies confirm the higher chance of fatality and severe disease in patients with comorbidities and add obesity as another risk factor. According to the American Center for Disease Control and Prevention obesity was present in 48.3% of the hospitalized patients with laboratory-confirmed COVID-19 (5). It was also reported that 89.3% of patients with COVID-19 registered in COVID-NET had other underlying conditions, most of them obesity-related: hypertension (49.7%), chronic lung disease (34.6%), diabetes mellitus (28.3%), and CVD (27.8%). Notably, among patients aged 50–64 years, obesity was most prevalent, followed by hypertension and diabetes mellitus. Among those aged >65 years, hypertension was most prevalent, followed by CVD and diabetes mellitus (5).

Furthermore, a study of 4,103 positively tested patients with COVID-19 in New York revealed more frequent hospitalization of patients with obesity (39.8 vs. 14.5%), CVD (44.6 vs. 16.4%), and diabetes (31.8 vs. 5.4%) than in patients without comorbidities (6).

## The Relationship Between Obesity and Unfavorable Outcome of COVID-19

Obesity is evidently associated with COVID-19 in terms of prevalence of symptomatic disease, disease severity and mortality (7, 8). Individuals with obesity have an approximately 3-fold increased risk of severe COVID-19 (7). Each unit increase in BMI was associated with a 12% increase in the risk of severe disease and obesity increased the need for invasive mechanical ventilation (7, 8). Furthermore, in patients below 60 years of age, who are generally considered to have a lower risk for severe disease, obesity appears to be a risk factor for hospital admission and need for critical care (9). Interestingly, similar associations were observed in other (viral) infections. During the Influenza A (H1N1) pandemic of 2009–2011, obesity was strongly associated with a worse disease outcome and death (10). Obesity also increases the duration of Influenza A virus shedding in adults, indicative of an impaired immune response that subsequently facilitates influenza transmission (11). Multiple mechanisms in obesity aggravate prognosis in COVID-19 (Table 1). Below we discuss the most important mechanisms known to date.

**Table 1:** Overview of mechanisms by which obesity contributes to worse prognosis of COVID-19.

Immune system	Chronic innate immune system activation
	Adaptive immune system dysregulation
	Lymphopenia
Endocrine and metabolic dysregulation in adipose tissue	Increased leptin secretion (pro-inflammatory)
	Decreased adiponectin secretion (anti-inflammatory)
	Hypoxia leading to low grade inflammation
Respiratory impairment	Reduced compliance, increased resistance
	Airway narrowing and collapse
	Reduced functional residual capacity
	Airway hyperresponsiveness
	Ventilation perfusion mismatch
Hemostasis	Enhanced prothrombotic state
Viral shedding	ACE2 expression on adipocytes
	Adipose tissue as potential reservoir for viruses
Comorbidities	Metabolic syndrome
	Type 2 Diabetes
	Cardiovascular disease, including hypertension
	Obstructive sleep apnea

### The Immune System

The immune system uses two sequential responses to react to disturbances in homeostasis, as seen in tissues under physiological or infectious stress. An initial rapid innate immune response, which has only broad specificity for the trigger is followed by a delayed, highly specific adaptive immune response (12). The latter generates long term target-specific immune memory. Both types of responses require specialized white blood cell types, such as granulocytes, macrophages, and natural killer cells that are innate immune populations, while different types of T- and B-lymphocytes constitute the adaptive immune cells. Although often designated distinct immune responses it should be realized that innate and adaptive responses are highly interdependent.

**Figure 1:**
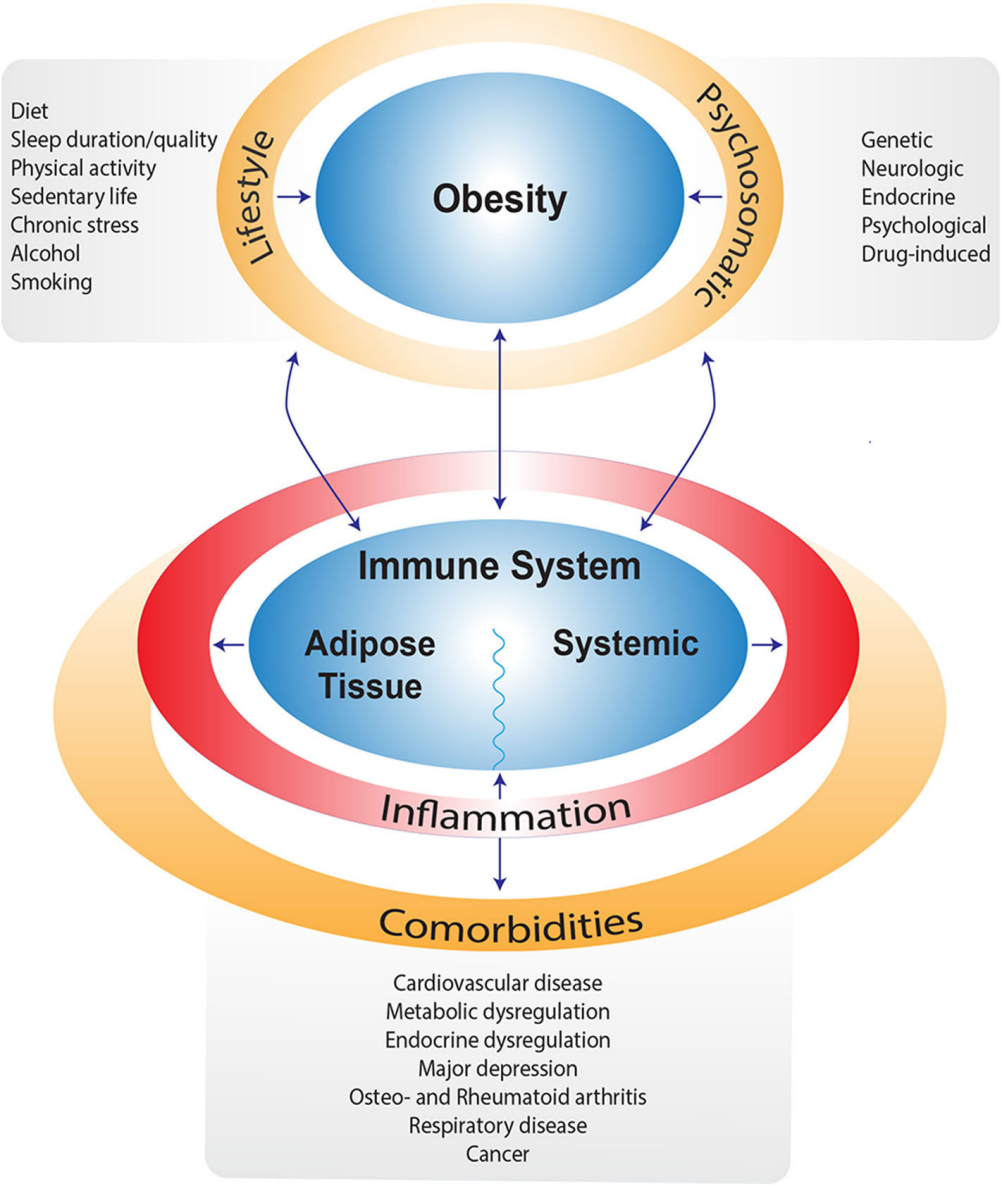
Schematic overview of factors contributing to obesity and/or impairing weight loss. Next to obesity, most of these factors are also directly related to alterations in the immune system leading to a pro-inflammatory state and subsequent comorbidities.

In obesity, both local and systemic immune alterations occur due to metabolic stress (13). In adipose tissue, the anti-inflammatory/immune regulatory primed immune cells (e.g., M2-type macrophages, regulatory T cells (Treg), T-helper (Th)2, type 2 innate lymphoid cells (ILC2) normally present in lean adipose tissue are replaced by increased numbers of pro-inflammatory primed immune cells (e.g., M1 macrophages, Th1, Th17, CD8^+^ T-cells) that secrete pro-inflammatory cytokines such as IL-1β, IL-6, IL-17, and IFN-γ. This may even be further enhanced by obesity-associated gut inflammation (13). In addition to local adipose tissue immune alterations, systemic immune adaptations are observed in obesity as well, including increased numbers of circulating (inflammatory) monocytes, neutrophils, Th1, Th17, Th22, decreased circulating Treg and elevated pro-inflammatory cytokine levels (14, 15). In concert, these changes lead to a pro-inflammatory state of the immune system in individuals with obesity, characterized by elevated cytokine levels, both locally in the adipose tissue as well as systemically, as depicted in Figure 1. This chronically elevated inflammatory state is thought to stimulate regulatory pathways, which in turn limit the response to an acute trigger such as SARS-CoV-2. This is exemplified by the hampered antiviral type I interferon response in individuals with obesity (16).

Besides systemic upregulation of pro-inflammatory cytokines, severe COVID-19 is associated with lymphopenia and T cell exhaustion (17). This may be related to functional depletion of the antigen-presenting dendritic cells due to viral infection, since they express both angiotensin-converting-enzyme 2 (ACE2) and the alternative viral receptor DC-SIGN (18). Substantial T-cell apoptotic death might then be caused by T-cell receptor engagement in the absence of appropriate co-stimulation, normally provided by dendritic cells. Moreover, SARS-CoV-2 hampers the function of CD4^+^ T cells and promotes excessive activation and possibly subsequent exhaustion of CD8^+^ T cells. Together, these perturbations of T cell subsets likely diminish host antiviral immunity (19). Obesity further worsens this condition as DC maturation appears to be hampered, which in itself already impacts the elicitation of appropriate T-cell responses (20).

### Endocrine and Metabolic Dysregulation in Adipose Tissue

Adipose tissue changes play an important role in obesity associated chronic systemic inflammation. Changes in adipokine secretion, fatty acid induced inflammation, oxidative stress, endoplasmic reticulum (ER) stress, and adipose tissue hypoxia are proposed mechanisms (21). Adipocytes secrete adipokines such as leptin and adiponectin, that can modulate the immune system (21, 22). In obesity, leptin secretion is increased. Leptin is pro-inflammatory and stimulates monocyte proliferation and differentiation into macrophages, modulates the activation of natural killer cells, and induces the production of pro-inflammatory cytokines such as TNFα, IL-6, or IL-12 (21). However, leptin inhibits anti-viral type I interferons (16). At the same time, the secretion of adiponectin, which has opposite immunomodulatory effects, is diminished. Adiponectin inhibits phagocytic activity and production of TNFα production, the synthesis of adhesion molecules, the formation of foam cells and stimulates the release of anti-inflammatory cytokines (e.g., IL-10). Nutrient excess and cell expansion in obesity can induce ER stress. ER stress is linked to the production of reactive oxygen species and the activation of inflammatory pathways (21). In addition, adipose tissue expansion in obesity eventually reaches a point where the capacity of local vasculature is insufficient leading to hypoxia. This hypoxic state aggravates the pro-inflammatory profile through further upregulation of leptin, downregulation of adiponectin, and increased production of pro-inflammatory cytokines by resident macrophages (21).

### Respiratory Impairment

Fat deposits in the mediastinum and abdominal cavity reduce compliance of the lungs, chest wall, and entire respiratory system. This likely contributes to respiratory symptoms of obesity such as wheeze, dyspnea, and orthopnea (23). The mechanical effects of obesity produce airway narrowing and closure, increased respiratory system resistance, reduced functional residual capacity, airway hyperresponsiveness, and a ventilation perfusion mismatch, all of which decrease lung function (23). Thus, altered lung function due to mechanical stress probably contributes to the worse prognosis in patients with obesity and COVID-19.

### Hemostasis

Obesity is independently associated with both arterial and venous thrombotic events (24). Related mechanisms are increased levels of clotting factors (tissue factor, Factor VII, Factor VIII, and plasminogen activator inhibitor) inflammation and endothelial dysfunction (24). COVID-19 in its severe form is also associated with both serious systemic inflammation and a prothrombotic state, as reflected by a significant increase in fibrinogen and D-dimer levels and high rates of severe pulmonary embolism that forecast a poor prognosis (25). This could constitute another link between obesity and worse outcome in COVID-19 but more research is required to confirm this notion.

### ACE2 Receptor, Viral Reservoir and Lymphopenia

Other potential mechanisms through which obesity may lead to worse COVID-19 prognosis are high ACE2 receptor expression and comorbidities. Expression of ACE2—the functional receptor for SARS-CoV-2 and other corona viruses—is upregulated in adipocytes of patients with obesity and diabetes, which turns adipose tissue into a potential target and viral reservoir (11). Potentially, this could result in prolonged viral shedding by adipose tissue, with extended activation of local “preactivated” immune responses and resident cytokine signaling pathways. However, currently, there is no evidence yet for direct SARS-CoV-2 infection of adipose tissue.

### Comorbidities

Comorbidities associated with obesity have become major health problems in patients that have been admitted to the hospital (8). Below, we discuss the influence of metabolic syndrome, T2D, CVD, and obstructive sleep apnea (OSA) on the immune system. Obesity associated insulin resistance drives the metabolic syndrome, a condition characterized by a clustering of three or more of the following: central adiposity, elevated blood glucose, elevated plasma triglycerides, elevated blood pressure, and low plasma high density lipoprotein (26). Components of the metabolic syndrome are associated with low-grade chronic inflammation. In metabolic syndrome, levels of leptin are increased aggravating leptin resistance enhancing chronic inflammation. Also, circulating adiponectin levels are decreased in metabolic syndrome, which impairs the resolution of inflammation. In itself, metabolic syndrome increases the risk of T2D and CVD by 2- and 5-fold, respectively (26).

The presence of T2D aggravates the prognosis in patients with COVID-19 (27, 28). It is associated with an approximately 2-fold increase of severe/critical COVID-19 illness, after adjustment for age, sex, obesity, hypertension, and smoking (28). Also, higher ICU admission rates and mortality risk were reported in patients with diabetes and COVID-19 (29). The hazard ratio of death was 1.53 in patients with diabetes after adjustment for age, sex, hypertension, CVD, and cerebrovascular disease (27). Notably, SARS-CoV-1-, MERS-, and influenza-infections are also marked by more severe symptoms in people with diabetes (27). Metabolic disruption, low grade systemic inflammation, and an unbalanced immune response may explain the more serious disease course of infections in T2D (28). This is further discussed in the Diet section.

CVD is a common comorbidity in COVID-19 (5). We did not find any literature that specifically explored the relationship between CVD and the capacity to respond to infectious challenges. Also, hypertension is often reported as a highly frequent comorbidity in severely ill patients with COVID-19 (5). Studies reveal higher ICU admission rates and higher mortality rates in patients with COVID-19 with hypertension (30). However, these data should be interpreted with caution as age and CVD may be important confounders (31). To date, there is no conclusive evidence for an independent causal link between hypertension and increased susceptibility for infectious diseases. Nonetheless, it should be realized that inflammatory conditions caused by innate and adaptive immune responses contribute to, or may even underlie alterations in vasculature, kidney, and the regulating sympathetic nervous system, which together cause hypertension (32).

It is believed that the underlying presence of OSA facilitates susceptibility to SARS-CoV-2 infection, and increases the risk for severe COVID-19 and the overall mortality of the disease (33). The CORONADO study shows that treated OSA is independently associated with risk of death by day 7 (34). Research suggests that when controlling for obesity, the presence of OSA is associated with decreased lung function, decreased lung-transfer factor for carbon monoxide, and, importantly, increased lung inflammation (35). Obesity plays a pivotal role in the pathogenesis of OSA and weight loss of approximately 7–11% leads to clinically significant and meaningful improvement of OSA (36). Lifestyle modification with reduced calorie intake and increased physical activity forms the foundation of all weight loss interventions and seems to have weight-independent benefits in OSA (36). Below we discuss the potential benefits of lifestyle adaptations in light of COVID-19 and its underlying risk factors.

## The Influence of Lifestyle Factors on the Immune System

When discussing the relation between obesity and the severity of COVID-19, it is important to emphasize that obesity is a complex disease. Next to societal and lifestyle factors, numerous other factors contribute to weight gain or impair weight loss. For example, medication may promote weight gain, as well as endocrine or hypothalamic disease, genetic syndromes or psychiatric conditions (37). It is counterproductive to blame people for their obesity or for severe progression of COVID-19. The obesity stigma is a serious problem in this context, and guiding people in their attempt to lose weight in a respectful way is of crucial importance (38, 39). Furthermore, when intensive lifestyle intervention turns out to be insufficient to lose weight, other obesity treatments such as pharmacotherapy or bariatric surgery should be considered to attain a healthier weight (40).

The American Endocrine Society and the European Association for the Study of Obesity both include lifestyle interventions, bariatric surgery and pharmacotherapy in recommended weight reduction strategies for people with obesity (41, 42). Besides weight reduction effects, bariatric surgery and pharmacotherapy have also been shown to positively affect the immune system in obese patients (43–46). The rate of weight loss for optimal immune system function is yet unclear. It is known that relatively fast weight reduction through bariatric surgery as well as more gradual weight loss through lifestyle modification both improve immune system markers (44, 47). On the other hand, severe caloric restriction, as well as malnutrition, reduces immunity and renders one more susceptible for disease (48, 49). Nevertheless, the negative impact of obesity on general health and responses to infections, including COVID-19 as discussed above, warrants vigorous lifestyle intervention (47).

**Figure 2:**
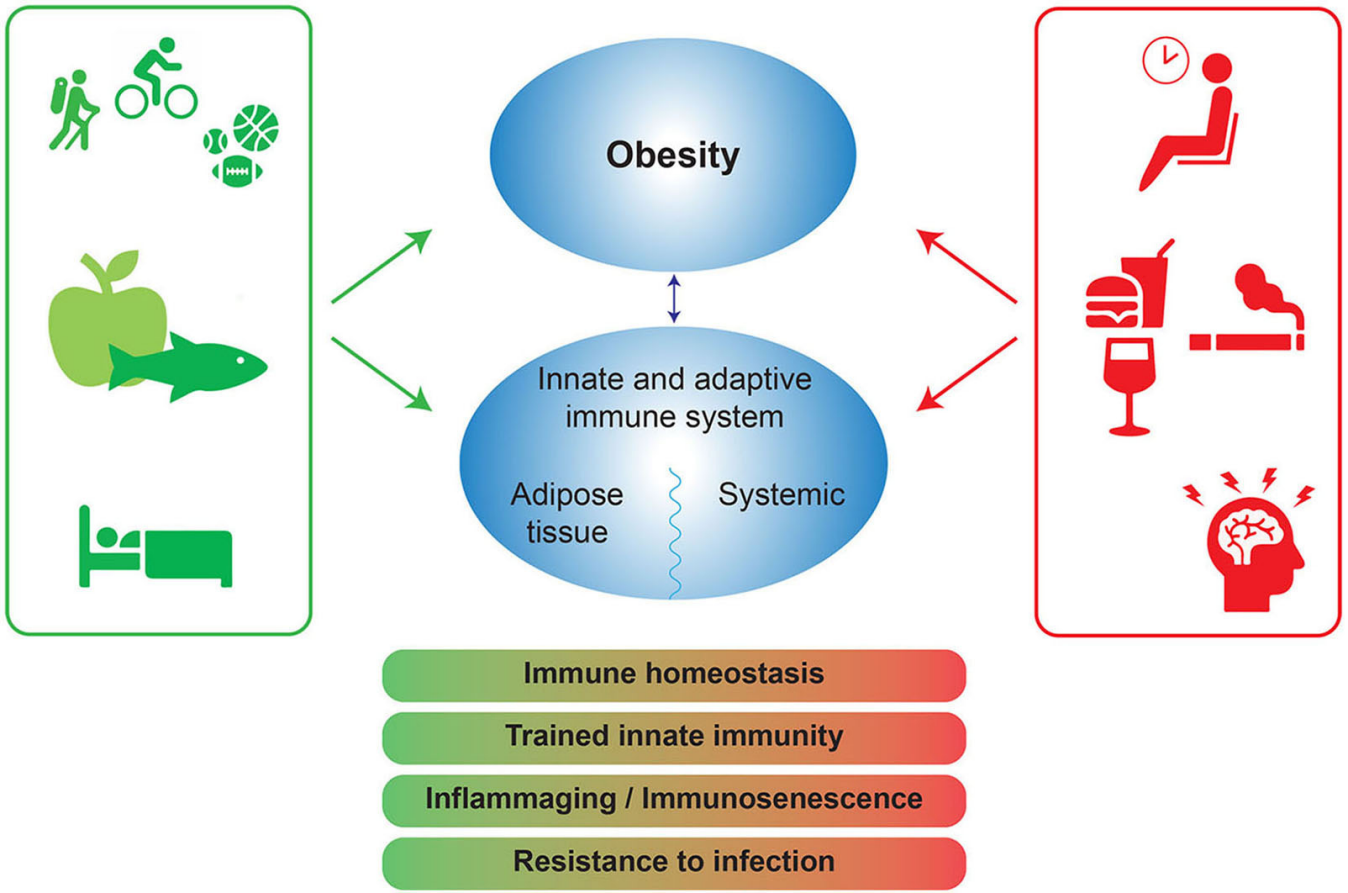
Positive and negative lifestyle factors influence obesity and the immune system. The negative lifestyle factors (red) can lead to a disbalance in immune homeostasis, trained innate immunity, inflammaging, and a decrease in infection resistance. Positive factors (green) can protect immune homeostasis and potentially increase resistance to infection. Trained innate immunity: long term functional reprogramming of innate immune cells, which is evoked by exogenous or endogenous stimuli and which leads to a usually enhanced response toward a second challenge after return to a non-activated state. Inflammaging: chronic low-grade inflammation that occurs with aging and is characterized by dysregulated inflammatory responses, in general resulting in increased inflammatory responses and diminished responses to pathogens like influenza or SARS-CoV-2.

Lifestyle features (nutrition, physical activity, sleep, stress, alcohol, smoking) interact with genetics to disrupt physiology and cause disease (50). In this context, a low grade inflammatory process appears to play a central role in many distinct chronic non-communicable disorders (51). Since lifestyle is consequently at the root of these diseases, it follows that lifestyle intervention should be an integral part of their treatment. Accordingly, a recent study shows that lifestyle intervention can partly reverse the pro-inflammatory phenotype associated with obesity (47). Even though the decline in pro-inflammatory markers was correlated with a decline of waist circumference and markers of metabolic syndrome, metabolic improvement was not required for immunological recovery. This suggests that lifestyle factors can directly affect the function of the immune system. Below we discuss the influence of diet, physical activity, stress, smoking, alcohol and sleep on the immune system as depicted in Figure 2.

### Diet

In affluent countries, chronic overeating of diets comprising a high proportion of ultra-processed energy-dense and nutrient-poor foods and drinks induces systemic low-grade inflammation, also referred to as “metaflammation” (52). These Western diet-components are thought to induce so-called “trained innate immunity,” i.e., the functional reprogramming of innate immune cells by exogenous or endogenous stimuli. Training leads to a more vigorous response toward a second activating challenge, and this condition may be retained for a long period. This innate memory gives rise to an elevated inflammatory set point of innate immune cells, which is mediated through alterations in their progenitor cells (53). Exposure to adverse metabolic stimuli induces epigenetic changes during early development of these immature innate immune cells, which facilitate the expression of inflammatory genes. The mechanisms explaining inappropriate adaptive immune activation against pathogens in the presence of enhanced innate-driven inflammation are far from clear at present. They might include immune suppression by activated myeloid cells or regulatory T- and B-cells as well as inappropriate skewing of adaptive responses (54).

The enhanced inflammatory set point in obesity is reminiscent of changes occurring during aging, which is also characterized by increase of inflammatory parameters and designated as “inflammaging” (55). In fact, alterations in DNA methylation in blood cells and other tissues can be used as epigenetic marker of biological age, the ‘epigenetic clock' (56). Lifestyle-related risk factors, including obesity and smoking, accelerate epigenetic aging (55). Moreover, obesity may promote thymic involution, leading to diminished output of naïve T-lymphocytes, which accelerates immunosenescence (57).

Dampening inflammation by adequate intake of fruit and vegetables is essential for prevention of chronic metabolic diseases, and sufficient consumption thereof is reflected by inhibition of epigenetic aging (55). Many fruits and vegetables, e.g., tomatoes, garlic, berries, apples, broccoli, grapes and olives, have anti-inflammatory effects (58). A number of vitamins (A, B6, B12, folate, C, D, and E) and trace elements (zinc, copper, selenium, iron) have been demonstrated to play important and complementary roles in supporting both the innate and adaptive immune systems (59). Foods such as nuts, meat, eggs, shellfish, and wholegrains are good sources of many of these trace elements. In addition, omega-3 fatty acids derived from fatty fish have anti-inflammatory action (59). There is evidence that omega-3 fatty acids can control the cytokine storm in acute respiratory distress syndrome (ARDS) (60). Such a diet is consistent with current healthy eating guidelines (60).

Foods containing probiotic and prebiotic substances can maintain a healthy microbiota, which can also benefit the immune system (60). The gut microbiota plays a role in educating and regulating the immune system. Gut dysbiosis is a feature of obesity, chronic disease and many infectious diseases, and has been described in COVID-19. Dietary approaches to achieve a healthy microbiota can also benefit the immune system. Indeed, optimal nutrient intake promotes optimal immune function and may limit the impact of novel, more virulent pathogenic viruses (59). It must be emphasized that severe caloric restriction, as well as malnutrition, reduces immunity and renders one more susceptible for disease (48, 49). Extreme and prolonged reduction of caloric intake or crash diets are therefore strongly discouraged.

Experimental evidence of the effects of diet in relation to COVID-19 is still lacking. However, it is generally accepted that a diet rich in vegetables, fruit, nuts, legumes, fish, and ‘healthy' dietary fats is associated with a lower risk of chronic non-communicable diseases as well as improved immune function (61). A range of nutrients or bioactive compounds has been proposed to explain this association. Examples are polyphenols, vitamin D, n-3 PUFA in autoimmune and inflammatory disorders, and vitamin D, vitamin E, zinc, and probiotics in reduction of infection (62). A balanced whole foods version of a vegetarian or Mediterranean diet fits this description. Conversely, a diet that is rich in energy-dense, nutrient-poor foods (i.e., consisting of ultra-processed foods with high levels of refined carbohydrates, added sugar and fats) is related to an increased risk of non-communicable diseases and impaired immune function (63). Moreover, a recent study found that ultra-processed food stimulated additional intake of approximately 500 kcal/day when compared to content-matched unprocessed diet (64).

### Physical Activity

Similar to the Western diet, a sedentary lifestyle is associated with abdominal adiposity, a pro-inflammatory state and increased risk of infection (65). Besides facilitating weight loss, regular physical activity decreases the activation of the immune system and improves immunosurveillance and immunocompetence (65). Regular exercise may limit or delay inflammaging (66). Subsequently, a physically active lifestyle reduces the risk of contracting a range of communicable diseases including viral and bacterial infections (65, 66).

The near-daily practice of moderate intensity exercise (e.g., walking or cycling) reduces upper respiratory tract infection symptom days by 40–50% (67). There is a dose-response relationship between the intensity of regular exercise and the risk for infection (65). Exercise intensity is commonly expressed as the Metabolic Equivalent of Task (MET) which is the ratio of a person's working metabolic rate relatively to their resting metabolic rate. Light to moderate exercise (MET 2–5.9, e.g., walking, vacuum cleaning or yoga) is better than inactivity (MET <2, e.g., writing or desk work) but vigorous exercise (MET 6–8.7, e.g., running, basketball or swimming) is even better. On similar note, short periods of maximum intensity training (High-intensity interval training or HIIT) seem to be a valuable addition to moderate-intensity continuous training in terms of enhancing cardiorespiratory fitness, metabolic hemostasis, vascular function and immune system function (68, 69). However, prolonged and intensive or (near) maximal intensity exercise is associated with an increased illness risk (67).

Substantial reduction in acute respiratory infection illness is detectable as soon as after 8 weeks of moderate intensity exercise (70). Most studies used a moderate intensity program of 30–45 min/day for 5 days/ week (67). This is in line with the Global recommendations on Physical Activity for Health on which most national guidelines are based (71). Thus, adoption of a physically active lifestyle by a substantial percentage of the population would probably curtail infectious pandemics such as COVID-19 and non-infectious pandemics such as obesity (65).

### Stress

Different emotional states, or exposure to psychological stressors, are associated with enhanced susceptibility to, or increased severity of disease through nervous system-induced alterations in innate and adaptive immunity (72). A main component of the stress response is cortisol, which production and secretion are regulated by the hypothalamus-pituitary-adrenal axis (73). Cortisol has many functions in the human body, such as mediating the stress response, and regulating metabolism as well as, inflammatory-, and immune function (73). Cortisol is ordinarily anti-inflammatory and confines the immune response, but chronic hypercortisolemia can lead to resistance of the immune system. The accumulation of stress hormones gives rise to an increased production of inflammatory cytokines that compromises the immune response (74). It has been demonstrated that individuals reporting higher levels of stress are more likely to develop clinical symptoms during experimental respiratory viral infection (72). Behavioral interventions targeted at alleviating stress, promoting heightened states of relaxation and encouraging moderate physical activity have been shown to bolster anti-viral immune responses and decrease markers of inflammation (75). A meta-analysis focusing on psychological interventions to alleviate stress showed positive effects on the immune system. It even suggested a positive relation between the frequency of self-reported of stress-reducing practice and a more adequate functioning of the immune system (76). Besides the impact on inflammatory markers, stress also promotes other negative lifestyle factors such as unhealthy eating and poor sleeping quality (77).

### Smoking

Nicotine and other tobacco components have several pro- as well as anti-inflammatory effects, and thus contribute to an altered immune set point in smokers (78). Current or former smoking is a risk factor for severe disease in patients with COVID-19. Smokers appear to have a 40% higher chance of developing severe symptoms, and a 140% higher chance of being admitted to an ICU, need mechanical ventilation or die as compared to non-smokers (79). A similar observation was made in patients with influenza (80). Smokers were over 5 times more likely to develop laboratory-confirmed influenza, and 34% more likely to develop influenza-like illness than non-smokers. Importantly, smoking cessation recovers airway ciliary clearance and immune function as early as 1 month after the intervention (81). Thus, smoking cessation is strongly encouraged as a public health measure to limit the global impact of COVID-19 (81).

### Alcohol

Alcohol consumption affects the number, survival and function of both innate and adaptive immune cells, thereby interfering with immune responses (82). Consumption of high doses can directly suppress a wide range of immune responses, and alcohol abuse is associated with an increased incidence of infectious disease (83). For instance, alcoholics are highly susceptible to respiratory pathogens and lung injury, and have a 2–4 fold increased risk of ARDS (84). Furthermore, alcohol consumption increases the risk of community-acquired pneumonia in adults (85). A dose-response relationship is observed, indicating that the risk for pneumonia increases by 8% for every 10–20 g (one unit of alcohol) higher daily alcohol intake. Surprisingly, even a single episode of binge drinking can have measurable effects on the immune system, inducing a transient pro-inflammatory state within the first 20 min after alcohol ingestion, followed by an anti-inflammatory state 2–5 h after alcohol ingestion (82). Also in animal models of pulmonary infections, alcohol administration is associated with adverse clinical parameters and increased lung damage (82). In many cultures, drinking alcohol is socially accepted and integrated in daily life. Increased awareness of the negative effects of alcohol and making moderation or abstinence the default choice are of paramount importance for public health.

### Sleep

It is suggested that both circadian rhythm and sleep significantly modify the susceptibility to SARS-CoV-2 infection as well as the overall clinical manifestations of COVID-19 (86). This relates to an interdependent balance maintained between the circadian clock, the intestinal microbiota and immune system activity (87). Naturally occurring sleep acutely enhances the expression of cytokines such as IL-12 and IFN-γ that regulate anti-viral immune defense mechanisms (88). Also, sleep might improve immunological memory formation by boosting the number of antigen presenting cells and CD4+ T-cells (89). The central nervous system seems to use the metabolically quiescent period of sleep to regulate both innate and antiviral immune responses (88). Sleep deprivation leads to decreased lymphocyte blastogenesis and natural killer cell activity, and upregulates IL-1 and IL-2 (90). However, sleep also increases IL-2 production by T-cells (88). Short habitual sleep (<6 h) is associated in humans with reduced life span, increased vulnerability to viral infection and reduced antibody titers after vaccination (91). In addition, short-term sleep deprivation prior to vaccination appears to negatively impact antibody titers after influenza vaccination (92). In rodents, sleep disturbances reduces influenza vaccine efficacy (93). When attempting to improve on sleep quality to boost the immune system, paucity of evidence prevents strong recommendations. However, a key point is that in current times circadian rhythm and sleep disruption frequently occur through shift work, nighttime light exposure and social jet lag (91). Morin et al. provide a condense overview of recommendations to improve sleep quality, especially during the COVID-19 pandemic (Table 2) (94). Further research could explore the effects of these recommendations on immunity and susceptibility to infections.

**Table 2:** Strategies to manage sleep disturbances during the COVID-19 pandemic.

1. Make sleep a priority and reserve at least 7–8 h per night for sleep.
2. Keep a regular schedule for sleep, meals, work, and social contacts. These activities are important time cues that help maintain entrainment of one's biological clock.
3. Get as much daylight exposure as possible (turn on the lights, open the curtains, go outside if possible) to regulate sleep-wake and circadian rhythms.
4. Avoid using electronics (cellular, tablets) in the bed or bedroom or near bedtime.
5. Allow at least 1 h to unwind before bedtime.
6. If sleep does not come within 15–20 min, go to another room and engage in quiet activities (reading), and return to bed only when sleep is imminent.
7. Reserve the bed and bedroom for sleep and sex only. This is not the place to worry, problem-solve, or plan the next day.
8. Get up at the same time every morning, regardless of the amount of sleep. While it may be tempting to sleep in, particularly when there is no obligation to be at work, it is best to arise at the same time to keep a regular sleep-wake schedule.
9. Although it is better to avoid napping for someone with insomnia, napping is beneficial for sleep-deprived people. Older adults, without sleep problems, may also benefit from a short catnap (15–20 min) around mid-day.
10. Keep in mind that short bouts of insomnia is a normal reaction to stressful life events, but when sleep difficulties occur several nights per week, take action and seek professional help to prevent acute insomnia from turning into chronic insomnia.

*Adopted from Morin et al. (94)*.

## An Upstream Environmental Approach Toward Healthier Lifestyles is Needed

Thus, we know that obesity, chronic disease and their underlying causes impair immune function and leave individuals more vulnerable to infection. These underlying causes include behavioral and modifiable factors such as an unhealthy diet, inadequate physical activity, chronic stress, use of alcohol and tobacco and suboptimal sleep quality (Table 3). To improve on all these aspects of health, multiple methods can be used. We make a case for health education, choice architecture, and regulation on local, national, and international levels.

**Table 3:** Aspects of lifestyle to be addressed to improve immune function.

Diet	• Increase the intake of healthy foods (e.g., vegetables, fruits, nuts, legumes, whole grains, and fish)
	• Decrease the intake of unhealthy foods and drinks (e.g., ultra-processed, refined carbohydrates, added sugars, and fats)
Physical activity	• Increase adherence to (inter)national physical activity guidelines
	• Decrease sedentary behavior
Stress	• Increase active relaxation
	• Decrease chronic stress (e.g., in studies, jobs, and social life)
	• Increase skills and opportunities to cope with chronic stress
Smoking and alcohol	• Decrease the marketing, sale and use of (legal) addictive substances such as tobacco and alcohol
	• Provide help for those addicted
Sleep	• Increase sleeping quality

Undeniably, widespread health education is an important aspect of reaching these lifestyle goals. This challenge is a shared responsibility of healthcare professionals, governments, the private sector, and civil society. Healthcare organizations and professionals should advocate the use of lifestyle as therapy in its own right, of equal importance to medication and interventions. Lifestyle modification should be an integral part of chronic disease management. National and local policies should address social determinants of health and health inequalities (95).

Extra attention should be given to personal health and lifestyle factors in periods of complete or partial lockdown. Lockdown periods lead to increases in sedentary behavior (unhealthy) food consumption, alcohol consumption, tobacco use, stress, and decreased well-being (96–98). Distressingly, increased food consumption and snacking are more prevalent in people with overweight or obesity. Even 2 weeks of physical inactivity and a positive energy balance can lead to reduced insulin sensitivity, higher total body fat, and a proinflammatory state (99). People should be aware of the influence of lockdown on their daily habits and be guided to eat healthy, exercise adequately, and find help for psychosocial adversities. We conclude that advocating home-confinement should be accompanied by stressing the need to maintain a healthy lifestyle.

Besides health education, we need to realize that our environment influences our choices substantially. Choices are made by means of two cognitive processes: the reflective and the impulsive system. The former is rational and conscious, the latter is quick, automatic, and subconscious (100). In the context of the current environment, we make approximately 219 decisions in regard to food per day, of which 90% subconsciously (101). Default options such as easy availability of unhealthy foods significantly influence our choices in a negative way (102). Thus, in order to promote healthy choices we need to create an environment in which the default option is a healthy option.

In many parts of the world people currently live in an obesogenic environment (103). In recent decades most societies increasingly offer an overabundance of cheap and convenient energy-dense and nutrient-poor foods and drinks, which contribute to adverse dietary choices. Intensive marketing of ultra-processed foods only makes matters worse. Moreover, many of the current environments promote sedentary behavior and discourage physical activity (104). As a society, we need to promote a healthy lifestyle, and thereby decrease rates of obesity and improve immune function. To do so, we need to reconstruct our living environment so as to facilitate and promote healthy dietary choices, physical activity, stress relieve, and regular sleep (105). Changing choice architecture can be done by changing food positioning in supermarkets, schools, or workplaces (106, 107). Another example is using built environment to increase physical activity and active transport (108).

In addition to choice architecture, creating healthy communities requires regulation by governments aimed at protecting society from the commercial determinants of chronic non-communicable diseases (109). This includes regulating the sale and promotion of unhealthy commodities such as tobacco, alcohol, and ultra-processed food and drinks (109). Strong leadership is essential to resist attempts by powerful organizations with vested interests (e.g., the tobacco, food, and alcohol industries) to undermine the development and implementation of effective policies and laws (110). Evidence-based approaches such as legislation, regulation, taxation, pricing, ban, and restriction of advertising and sponsorship should be introduced to reduce consumption of unhealthy commodities (109). All in all, the campaign for healthier lifestyle includes, but is not limited to, aspects on health education, environmental changes, and regulatory actions (Table 4).

**Table 4:** Facets of the campaign for healthier lifestyles.

Health education	• Public
	• Healthcare professionals
	• Policy makers
	• Extra attention during lockdown
Environment	• Supermarkets, Schools, Workplaces
	∘ Food positioning (106)
	• Built environment
	∘ Physical activity and active transport (108)
Regulation	• Taxation, pricing, ban, and restriction of advertising and sponsorship (109)

## Conclusions

COVID-19 has significant impact on public health. Obesity, chronic disease, and various lifestyle components increase the severity of the disease course, the appeal to health care, and the mortality rate of SARS-CoV-2 infections. The harmful impact of these factors on the immune system probably explains the worse outcome to a large extent. Conditions associated with low-grade systemic inflammation, such as obesity, metabolic syndrome, and old age, hamper an optimal and timely response by the immune system to an infectious challenge like SARS-CoV-2. Several Western diet components train innate immune cells so as to adopt a more pro-inflammatory set point, which enigmatically diminishes their capacity to activate adequate adaptive immunity required for an effective battle against pathogens.

Adapting lifestyle on a global scale can most likely enhance population resistance to viral and other infections. However, making healthy choices is not as easy as knowing what to choose. In this context it is important to be aware of the existing obesity stigma and to counteract it. Obesity and related cardiometabolic diseases are not only a problem of the individual, but a burden for society as well. These metabolic disorders are primarily caused by environmental and societal factors, in concert with genetic and other individual (biological) characteristics. Reconstruction of our living environment so as to promote healthier lifestyles is of paramount importance to both prevent and treat obesity and related disorders. Obviously, a collective effort is required to get this done. Healthcare institutions, governments, the private sector, and (creative) citizens have to put their heads together to map out the route to a healthy future. Together we can change our lifestyle-choice architecture to make healthy living the norm, and thereby improve immune function to be better prepared for the next viral pandemic.

## Author Contributions

DF wrote and performed the literature searches, analyzed the results, selected relevant articles for this manuscript, and drafted the concept version of the manuscript. ER took the lead in supervising the creation process, provided content related to obesity, and gave feedback on every draft. DA, PL, WD, HP, and JS provided feedback during the creation process. Specifically on their fields of expertise but also on the manuscript as a whole. WD and PL designed both figures adopted in the manuscript. All authors provided them with feedback. All in all, DA, PL, WD, HP, and JS contributed roughly equally to the formation of the manuscript.

## Conflict of Interest

The authors declare that the research was conducted in the absence of any commercial or financial relationships that could be construed as a potential conflict of interest.
