# Supplementary material for: The Impact of Obesity and Lifestyle on the Immune System and Susceptibility to Infections Such as COVID-19
Source: Front Nutr. 2020 Nov 19;7:597600. doi: 10.3389/fnut.2020.597600 (PMC7711810; doi:10.3389/fnut.2020.597600)
Supplement: Supplementary file 1 [file Table_1.DOCX]

Search strategies

Strategy 1

Combining the search subjects “Coronavirus” and “diabetes/hypertension/obesity/cardiovascular disease/metabolic syndrome”.

Yielding 1132 articles on April 29^th^.

(“Coronavirus”[ti] OR “COVID-19”[ti] OR “Corona” [ti] OR "COVID-19" [Supplementary Concept] OR “2019 novel coronavirus disease” [ti] OR “COVID19” [ti] OR “COVID-19 pandemic” [ti] OR“SARS-CoV-2 infection” [ti] OR “2019 novel coronavirus infection” [ti] OR“2019-nCoV infection” [ti] OR “coronavirus disease 2019” [ti] OR “coronavirus disease-19” [ti] OR “2019-nCoV disease” [ti] OR “COVID-19 virus infection” [ti]) AND ("Diabetes Mellitus, Type 2"[Mesh] OR "Hypertension"[Mesh] OR "Cardiovascular Diseases"[Mesh] OR "Obesity"[Mesh] OR “Diabetes mellitus, type II” [tw] OR “Diabetes mellitus” [tw] OR “Type 2 diabetes mellitus” [tw] OR “Diabetes” [tw] OR “Diabetes type 2” [tw] OR “Type 2 diabetes” [tw] OR “hypertension “ [tw] OR “high blood pressure” [tw] OR “high blood pressures” [tw] OR “blood pressure, high” [tw] OR “blood pressures, high” [tw] OR “cardiovascular disease” [tw] OR “cardiovascular diseases” [tw] OR “obesity” [tw] OR “obese” [tw] OR “morbidly obese” [tw] OR “morbid obesity” [tw])

Based on title and abstract, 17 articles were selected and analysed.

## Strategy 2

Combining “Immune system” and “diabetes/hypertension/obesity/cardiovascular disease/metabolic syndrome”.

Yielding 1364 articles on May 17. 2020.

((("Metabolic Syndrome"[majr] OR "metabolic syndrome"[ti] OR "Obesity"[majr] OR "obesity"[ti] OR "obese"[ti] OR "Overweight"[majr] OR "overweight"[ti] OR "Diabetes Mellitus"[majr] OR "diabetes"[ti] OR diabetic*[ti] OR "Hypertension"[majr] OR "hypertension"[ti] OR "Cardiovascular Diseases"[majr:noexp] OR "Cardiovascular Diseases"[ti] OR "Cardiovascular Disease"[ti]) AND ("Immune System"[majr:noexp] OR "immune system"[tiab] OR "Immune response"[ti] OR immunosuppress*[ti] OR "immunity"[ti] OR infection*[ti] OR "Virus Diseases"[majr] OR "Respiratory Tract Infections"[majr] OR "Virus Disease"[ti] OR "Virus Infections"[ti] OR "Virus Infection"[ti] OR "Viral Diseases"[ti] OR "Viral Disease"[ti] OR "Viral Infections"[ti] OR "Viral Infection"[ti] OR "Influenza"[ti] OR ”COVID” [ti] OR ”Coronavirus”[ti] OR ”COVID-19” [ti] OR ”H1N1” [ti] OR ”dengue” [ti] OR ”flavivirus” [ti] OR virus*[ti]) AND (Impaired*[tiab] OR Dysfunction*[tiab] OR "risk of infection"[tiab] OR complication*[tiab] OR suscept*[tiab] OR "Disease Susceptibility"[mesh] OR "compromised"[tiab] OR "risk"[ti] OR "risks"[ti])) OR (("Metabolic Syndrome/immunology"[majr] OR "Obesity/immunology"[majr] OR "Overweight/immunology"[majr] OR "Diabetes Mellitus/immunology"[majr] OR "Hypertension/immunology"[majr] OR "Cardiovascular Diseases/immunology"[majr]) AND (Impaired*[tiab] OR Dysfunction*[tiab] OR "risk of infection"[tiab] OR complication*[tiab] OR suscept*[tiab] OR "Disease Susceptibility"[mesh] OR "compromised"[tiab] OR "risk"[ti] OR "risks"[ti]))) AND ("review"[ptyp] OR "review"[ti] OR "overview"[ti] OR "systematic"[sb] OR "Systematic Review"[ptyp] OR "Systematic Reviews as Topic"[Mesh] OR "Meta-Analysis"[ptyp] OR "Meta-Analysis as Topic"[Mesh] OR "Meta-Analysis"[tw] OR "Metaanalysis"[tw] OR jsubsetaim[text]) NOT ("human immunodeficiency virus" [tw]) NOT ("type 1 diabetes" [ti])

Based on title and abstract, 151 articles were selected and analysed.

## Strategy 3

Combining “immune system” and “lifestyle”.

Yielding 753 results on june 4^th^.

(("Life Style"[majr] OR "Life Style"[ti] OR "Lifestyle"[ti] OR "Life Styles"[ti] OR "Lifestyles"[ti] OR "Alcohol Drinking"[majr] OR "Alcohol consumption"[ti] OR "Drinking"[ti] OR "sedentary" [ti] OR "sleep"[ti] OR "Stress, Psychological"[majr]) AND ("Immune System"[majr:noexp] OR "immune system"[tiab] OR "Immune response"[ti] OR immunosuppress*[ti] OR "immunity"[ti] OR infection*[ti] OR "immune function"[ti] OR "immunometabolic"[ti] OR immune marker*[ti] OR immunological marker*[ti] OR "inflammation"[ti] OR "Virus Diseases"[majr] OR "Respiratory Tract Infections"[majr] OR "Virus Disease"[ti] OR "Virus Infections"[ti] OR "Virus Infection"[ti] OR "Viral Diseases"[ti] OR "Viral Disease"[ti] OR "Viral Infections"[ti] OR "Viral Infection"[ti] OR "Influenza"[ti] OR ”COVID” [ti] OR ”Coronavirus”[ti] OR ”COVID-19” [ti] OR ”H1N1” [ti] OR ”dengue” [ti] OR ”flavivirus” [ti] OR virus*[ti])) AND ("review"[ptyp] OR "review"[ti] OR "overview"[ti] OR "systematic"[sb] OR "Systematic Review"[ptyp] OR "Systematic Reviews as Topic"[Mesh] OR "Meta-Analysis"[ptyp] OR "Meta-Analysis as Topic"[Mesh] OR "Meta-Analysis"[tw] OR "Metaanalysis"[tw]) NOT ("human immunodeficiency virus"[ti] OR "HIV"[ti] OR "HIV Infections"[majr] OR "HIV Infections"[majr] OR "type 1 diabetes"[ti] OR "Diabetes Mellitus, Type 1"[majr] OR "lupus"[ti] OR "crohn"[ti] OR "Crohn's"[ti] OR "multiple sclerosis"[ti])

Based on title and abstract, 47 articles were selected and analysed.
